# An identity for the inscrutable *Homo habilis*

**DOI:** 10.1002/ar.70145

**Published:** 2026-01-24

**Authors:** Ian Tattersall

**Affiliations:** ^1^ Division of Anthropology American Museum of Natural History New York USA

By dint of incessant repetition, the species name *Homo habilis* has become firmly entrenched in the paleoanthropological lexicon. But what exactly does it denote? While the question is obvious, it has long been tricky to answer, because until recently most of us would have been hard put to come up with a satisfactory morphological (or indeed any other) definition for a species that has accreted new putative members over time as a matter of default, rather than of morphological coherence. The resulting difficulty of definition has actually been with us since the very beginning, because when Louis Leakey, Philip Tobias, and John Napier came up with the “handy man” moniker some 62 years ago (Leakey et al., 1964), they applied it to a rather miscellaneous assortment of dental and postcranial fossils from a variety of stratigraphic levels in Beds I and II at Tanzania's Olduvai Gorge that they grouped together, not on the basis of shared and unique apomorphies or even of firm taphonomic association, but instead to acknowledge Leakey's doctrinaire fealty to the notion of “Man the Toolmaker.” At the time, the venerable idea that humankind was defined by a behavior, the manufacture of tools, rather than by any morphological attribute(s), had recently been reinvigorated by Kenneth Oakley at book length (Oakley, 1961); and it was also, of course, deeply implicit in the choice of *Homo* as the genus of the Olduvai fossils. The discovery of OH7 and the other gracile fossils arbitrarily bundled into *Homo habilis* had actually come as an enormous relief to Leakey, following as it did a brief but unnerving period during which the uncomfortably “robust” OH5, nicknamed “Nutcracker Man” on account of its flat and enormous molar teeth, was the only obvious candidate for manufacturer of the Mode 1 tools that eroded out in abundance from the lowest strata of the Gorge.

Yet, as Pilbeam and Simons (1965) were quick to point out, the Bed I and lower Bed II Olduvai hominins attributed to *Homo habilis* represented a substantial variety in both age and morphology; and there was in addition widespread muttering to the effect that not enough “morphological space” existed between the new dental materials from Tanzania and the long‐established South African *Australopithecus africanus* to admit the new taxon (see Tattersall, 2009). As a result of such uncertainties it was not until after the discovery, in Kenya's East Turkana region, of the cranium KNM‐ER 1470 (Leakey, 1973) that discussion finally tilted in favor of *Homo habilis* as a real biological entity to be reckoned with—although the notion that ER 1470 represented a distinctive species of the genus *Homo* was basically driven by a new imperative: an endocranial volume considerably in excess of anything known from lower Olduvai.

Subsequent putative accretions to *Homo habilis* have included such candidates as the East Turkana cranium KNM‐ER 1813 (Howell, 1978), the fragmentary Olduvai and East Turkana skeletons OH 62 and ER 3735 (Johanson et al., 1987; Leakey et al., 1989), the Olduvai and Hadar palates OH 65 and NME‐AL 666 (Clarke, 2012; Rak et al., 1996), the Ethiopian LD 350‐1 Ledi‐Geraru partial mandible (Spoor et al., 2015), and the South African Stw 53 cranium (Hughes & Tobias, 1977). This motley assortment is notably varied in morphology, age, and body parts represented, and it gives *Homo habilis* a suspiciously heterogeneous and long‐lived hypodigm (approximately 2.8–1.6 Ma).

When Louis Leakey and his colleagues named the species *Homo habilis*, they specified as holotype the fossils that had been catalogued together as OH7. These consisted of a “mandible with dentition and the associated upper molar, parietals, and hand bones, of a single juvenile individual from site F.L.K.N.N.I, Olduvai Bed I” (Leakey et al., 1964, p. 8). Confusingly, other Bed I and lower Bed II specimens were named as paratypes; but under the rules of nomenclature any dental assignments to *Homo habilis* have necessarily to be made via comparison with the beautifully preserved lower dentition of OH7 (Figure 1). Until 2019 there was not much in the East African record to invite close comparison of this kind; but in that year Grine et al. (2019) reported the recovery, from 2.02 Ma sediments at Ileret in Kenya's East Turkana, of a series of isolated teeth that appeared to represent the full lower dentition of a young hominin individual that was designated KNM‐ER 64060 (Figure 1). In their initial report (and in implicit recognition of the difficulties that *Homo habilis* posed), Grine et al. (2019) simply assigned those teeth to “early *Homo* sp.”, noting that overall tooth crown size and various details of the premolars and molars aligned ER 64060 with the “early *Homo*” group rather than with the more microdont and locally slightly later *Homo erectus* (=*Homo ergaster*). They additionally observed that those isolated teeth had been found in a horizon that had also yielded “a series of postcranial bones … that may be associated with the dentition,” although “their separation on the surface of the site leaves some room for doubt” (Grine et al., 2019, p. 152).

**FIGURE 1:**
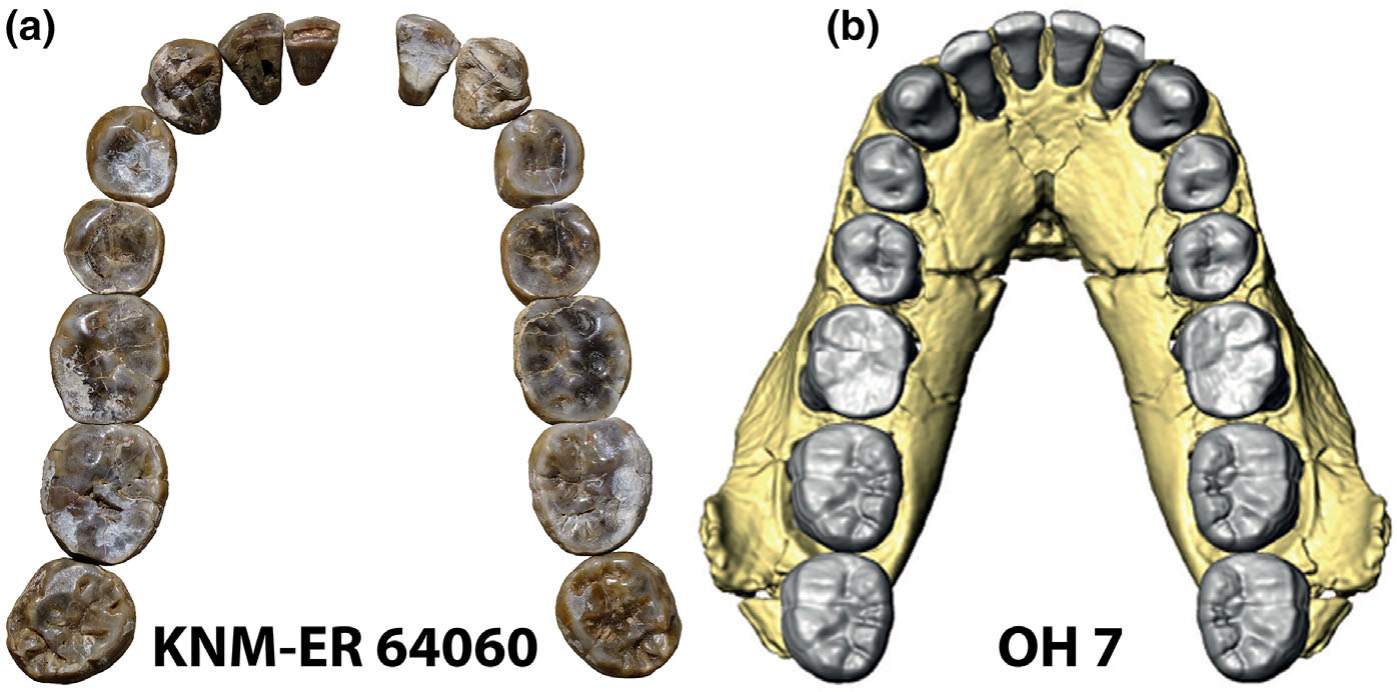
Comparison between (a) the isolated lower teeth of KNM‐ER 64060 (arrangement in anatomical position by Fred Grine) and (b) the virtual reconstruction of the crushed OH7 lower jaw by Spoor et al. (2015). To scale. Illustration courtesy of Heather Smith.

Grine and his coauthors have now clarified their initial interpretation by explicitly recognizing the ER 64060 teeth as those of *Homo habilis* (Grine et al., 2026). They provide only cursory justification for that taxonomic move, but there is little doubt that the close similarities in dental size and proportions between the OH7 and ER 64060 dentitions (Figure 1) suggest very strongly that the broadly penecontemporaneous Olduvai and Ileret materials do indeed represent individuals of the same species, by definition *Homo habilis*. As importantly, Grine and colleagues also offer compelling geochemical and taphonomic evidence to support the association of the ER 64060 teeth with the partial hominin skeleton that had been discovered nearby and given the identifier ER‐64061 (see also Present et al., 2024).

Although confined mostly to the upper limb (minus the hand) and bits of the pelvis, the 64061 skeleton tells us more about *Homo habilis* than any other of the very few and highly fragmentary postcrania that have been attributed to it, thereby justifying Grine and coauthors' claim that the ER 64060/64061 combination “very likely represents not only the most complete but also the oldest postcranial skeleton of early *Homo*” (Grine et al., 2026). The picture that emerges from Grine and colleagues' meticulous and beautifully illustrated descriptions and comparisons of ER 64061 (which appropriately acknowledge the many uncertainties its incompleteness imposes in exercises such as the estimation of body proportions) in many ways confirms, as well as extends, the results of earlier analyses of putatively *Homo habilis* postcranial fossils.

Johanson et al. (1987) described the highly fragmentary Olduvai OH 62 skeleton they attributed to *Homo habilis* as remarkably similar to the Ethiopian “Lucy” NME A.L. 288‐1 skeleton of *Australopithecus afarensis*, albeit with a more robust and even longer forelimb reminiscent in its proportions of African apes. Similarly, Haeusler and McHenry (2007) found that the ER 3735 partial skeleton, also believed to be that of *Homo habilis*, had chimpanzee‐like brachial proportions, with a longer and more robust forearm than that of *A. afarensis*. Grine and colleagues now report that something broadly similar was likely true for the arm of the ER 64061 individual, which “would have possessed a relatively long upper limb for the size of its lower limb” (Grine et al., 2026). When the authors put this together with tantalizing hints from other parts of the postcranial skeleton, a portrait of *Homo habilis* emerges that is remarkably archaic for a putative member of our own genus, and that contrasts very substantially with what we see in the ~1.6 Ma WT 15000 skeleton from Nariokotome in West Turkana that is nowadays taken as the prime exemplar of *Homo ergaster* (a.k.a. “early African *Homo erectus*”). That species was originally named from a distinctive and somewhat younger mandible with dentition (ER 992) from Ileret; and, in stark contrast to ER 64061, the Nariokotome skeleton exhibits broadly modern limb proportions.

Grine and colleagues describe ER 64061 as having a rather australopith‐like upper body, at least in preserved elements; and although they assert that the individual's “lower limb mechanics” were “more similar to *Homo* than australopiths” (Grine et al., 2026), the main evidence supporting this claim is a small ischial fragment that is “consistent with this fossil's attribution to *Homo* (cf. KNM‐WT 15000) only inasmuch as it differs from ischia of *Australopithecus* (e.g., AL 288‐1 and Sts 14)” (Grine et al., 2026). Indeed, the meager pelvic fragments of ER 64061 proved most useful in enabling a very modest (hence australopith‐like) body‐mass estimate of 30.7 kg, based principally on a rough approximation of femoral head diameter. What is more, the team very approximately estimated the stature of ER 64061 (principally from the arm bones, with all the many attendant uncertainties) at around 160 cm (5′ 2″). This is close to the top end of the australopith range, but well below even the lowest estimate of the adult height of the immature WT 15000 *Homo ergaster* individual (Graves et al., 2010).

Grine and colleagues' careful descriptions and cautious but probing analyses thus add up to a portrait of a *Homo habilis* that “retained more primitive proportions and was smaller in stature and mass than *Homo erectus*” (Grine et al., 2026). In combination with the earlier work they cite, and with the identity conferred by the dentition, this insight enables us finally to envisage *Homo habilis* as a morphologically definable and dynamically functioning entity: a recognizable actor in the hominin evolutionary play, rather than merely a wastebasket into which miscellaneous hominin fossils have been conveniently tossed because they were not self‐evidently something else. Partial as our current view of the species may necessarily be, *Homo habilis* emerges as a relatively diminutive early hominin toolmaker that possessed a remarkably archaic upper body skeleton and was presumably a significant interactor in the woodlands and bushlands of what are now Tanzania and Kenya (and maybe of Ethiopia too) during the period centering around 1.8–2.0 Ma.

Although its central aim is description and functional analysis, Grine and colleagues' contribution also helps greatly in the essential process of sorting out just how diverse in species early hominins were—a task that has for far too long been neglected in favor of concocting linearist or selectionist explanations for events in early human evolution. But did OH7 and ER 64060/1 really belong to our own genus *Homo*, as Louis Leakey so desperately wanted for the former? That question now emerges with enhanced urgency, because virtually everything the Grine group has established about them suggests that the Ileret fossils and their Olduvai conspecifics are descriptively more closely similar to members of *Australopithecus*, or at least to members of the clade to which *Australopithecus* belongs, than they are to anything that plausibly belongs to the genus defined by our own species *Homo sapiens*. As Wood and Collard (1999) pointed out, if the genus *Homo* is to have any workable definition, its members need to conform to certain basic criteria. Those benchmarks include that they be demonstrably more closely related to *Homo sapiens* than to the australopiths; that in body mass and proportions they should more closely resemble the former than the latter; and that they should show evidence of *Homo*‐like obligate bipedality and extended growth schedule. On the basis of the limited material known, the *habilis* fossils from Olduvai and Ileret fail to qualify as *Homo* on at least two and maybe all three of these counts. That exclusion contrasts starkly with the very morphologically distinct and only slightly younger Turkana materials, such as the skeleton WT 15000 and the cranium ER 3733, that are often assigned to *Homo ergaster* (or, as by Grine and colleagues, to African *Homo erectus*).

But does this necessarily mean that the relatively diminutive ER 64060/1 individual, with its postcanine megadonty and elongated arms, should be classified as *Australopithecus*? Of course not. It just means that it is excluded from *Homo*, although not necessarily from the larger *Homo* clade that findings at sites such as Ethiopia's Ledi‐Geraru (Villmoare et al., 2015, 2025) suggest may already have been diversifying, in parallel to the australopith one, as early as around 3 Ma (see also Spoor et al., 2015). Sadly, though, while most paleoanthropologists have moved beyond the absolutist unilinearism that Ernst Mayr inflicted upon them three‐quarters of a century ago (Mayr, 1950), the ornithologist's influence still lingers in the form of a persistent taxonomic minimalism that resists full recognition of hominin diversity by broadly holding that, if a Pleistocene hominin fossil is not *Australopithecus* (or *Paranthropus*), it must be *Homo*—or vice versa (Tattersall, 2022, 2024). This formula has simplified life for paleoanthropologists by allowing them to skip over the undoubted difficulties of alpha systematics; but it has led to the accumulation of a lot of very odd bedfellows within our genus.

We urgently need to move beyond this constricting mindset, especially since the message compellingly emerging from the rapidly expanding hominin fossil record is of a diversity that contrasts starkly with Mayr's strictly unilinear picture. Put another way, the speciation and evolutionary experimentation already evident in what is by now a very extensive hominin record makes it clear just how much the unfortunate Mayrian legacy still hinders our ability to make proper sense of our biological past. Heretical though the thought may be, we need more hominin genera than are presently admitted both to express and to organize the diversity that the burgeoning number of accepted fossil hominin species suggests is out there. Given the current paleoanthropological zeitgeist, it is entirely understandable that Grine and colleagues should wish to downplay the radical systematic implications of their study in a contribution that is more narrowly aimed at showing—very elegantly—just how much functional information and informed conjecture can be extracted from the fragmentary remains of an extinct hominin. But the systematic signal that is so clearly implicit in their conclusions is of equally vital importance.

## AUTHOR CONTRIBUTIONS

**Ian Tattersall:** Conceptualization; writing – review and editing.
